# Deep-brain imaging via epi-fluorescence Computational Cannula Microscopy

**DOI:** 10.1038/srep44791

**Published:** 2017-03-20

**Authors:** Ganghun Kim, Naveen Nagarajan, Elissa Pastuzyn, Kyle Jenks, Mario Capecchi, Jason Shepherd, Rajesh Menon

**Affiliations:** 1 Department of Electrical and Computer Engineering, University of Utah, Salt Lake City, UT 84112, USA; 2 Department of Human Genetics, University of Utah, Salt Lake City, UT 84112, USA; 3 Department of Neurobiology and Anatomy, University of Utah, Salt Lake City, UT 84112, USA

## Abstract

Here we demonstrate widefield (field diameter = 200 μm) fluorescence microscopy and video imaging inside the rodent brain at a depth of 2 mm using a simple surgical glass needle (cannula) of diameter 0.22 mm as the primary optical element. The cannula guides excitation light into the brain and the fluorescence signal out of the brain. Concomitant image-processing algorithms are utilized to convert the spatially scrambled images into fluorescent images and video. The small size of the cannula enables minimally invasive imaging, while the long length (>2 mm) allow for deep-brain imaging with no additional complexity in the optical system. Since no scanning is involved, widefield fluorescence video at the native frame rate of the camera can be achieved.

Imaging deep inside biological tissue such as the brain is vital for many applications. Multi-photon microscopy can achieve this for limited depths and at limited resolution. The multi-photon microscope works by scanning a focused laser beam throughout the volume of interest inside the specimen, in a manner similar to a scanning confocal microscope except that it uses multi-photon absorption phenomenon to excite fluorescent labels. Excitation at longer wavelengths (>780 nm) offers maximum penetration depths between 600 and 800 μm given 1 W average excitation power, which could increase to 1 mm when the laser is accompanied by a regenerative amplifier to produce stronger laser pulse^1^. However, this comes at the expense of poorer spatial resolution because the diffraction limit is dictated by the longer wavelength and by scattering in tissue. With three-photon microscopy, penetration depth of up to 1.2 mm was recently reported^2^. However, three- or multi-photon excitation is extremely inefficient due to the low absorption cross-section, which requires large excitation intensities leading to potential for photo-toxicity. Furthermore, many interesting biological features lie at depths greater than 1.2 mm from the surface of the brain such as the basal ganglia, hippocampus, and the hypothalamus^3^,^4^.

Microendoscopy is the general approach to image features deeper than those achievable with multi-photon imaging. In microendoscopy, a miniaturized optical probe is inserted into a region of interest in the brain. The probe transports optical signal in and out of the region. The most common example is a fiber bundle^5^,^6^,^7^,^8^. Unfortunately, fiber bundles tend to be rather large, potentially resulting in trauma for deep-brain imaging. Furthermore, resolution below ~3 μm is challenging due to cross-talk between adjacent cores^5^,^6^. Another approach for microendoscopy uses a miniaturized microscope with gradient index (GRIN) microlenses^9^,^10^,^11^. GRIN microlenses require a spatially varying refractive index, which is typically created by controlled doping of glass^12^. These lenses suffer from relatively large size, low efficiency, reduced field of view due to off-axis aberrations and high costs^12^,^13^,^14^. Current state of the art GRIN lens based microendoscopy offers 450 μm^2^ or 300 μm^2^ field of view when it is equipped with 1 mm or 0.5 mm diameter imaging probe, respectively, which corresponds to 25 or 45% effective imaging area relative to the probe size. In practice, the GRIN microlenses need to be used in conjunction with a regular microscope^9^,^10^, or a miniaturized microscope^11^, both of which significantly increase the complexity of the system. Various other designs have also been proposed or demonstrated as summarized in several review articles^15^,^16^. However, none of these approaches provide the combination of deep penetration, small size and low cost for widespread use in fluorescence imaging of the deep brain.

Here, we utilize a simple surgical glass needle, a cannula (diameter = 220 μm) as our primary imaging element and demonstrate fluorescent imaging and video from the mouse brain at depths as great as 2 mm with a field of view of 200 μm in diameter. Furthermore, we are able to image features of about 3.5 μm in size. Due to its small size and large effective imaging area, the cannula is able to achieve the comparable field of view as that obtained via much larger GRIN-lens probes but with significantly less tissue damage. Previously, we demonstrated this technique, which we call computational-cannula microscopy (CCM) for high-resolution, wide-field fluorescence microscopy of samples in air^17^,^18^. Here, we extend CCM to operate in an epi-illumination configuration, where the excitation beam is guided inside the sample by the cannula. The fluorescent signal from inside the tissue is guided to the outside by the same cannula, which is analogous to an epi-fluorescence microscope. Our approach is quite distinct from other methods that utilize a step-index fiber for micro-endoscopy^19^,^20^,^21^,^22^,^23^, since these require scanning or only operate on coherent signals. Consequently, we are able to achieve fast (at the native frame rate of the camera), widefield fluorescence imaging from deep regions of the mouse brain through a small glass cannula with minimal tissue damage.

The working principle of CCM has been described previously^17^,^18^ and is summarized in Fig. 1(a). A fluorescent point source at the distal end of the cannula creates a pattern on the proximal end. This pattern is highly sensitive to the precise location of the point source as indicated by the experimentally obtained images in Fig. 1(a). This is an example of a space-variant point-spread function (SV-PSF). In CCM, a calibration process is first performed that creates a map of such SV-PSFs using a point emitter, a fluorescent microsphere that is stepped across the field of view. The unknown object is treated as a linear combination of fluorescent point emitters. The resulting image on the proximal end of the cannula is then the linear combination of the corresponding SV-PSFs. Given this complex image, we apply a Tikhonov-regularization based algorithm to recover the details of the object^24^. Currently, it takes less than 0.2 s to compute each frame on a desktop computer (Dell XPS 8700, Intel Core i7-4790 3.6 GHz, 32GB RAM).

## Experiment

**Figure 1:**
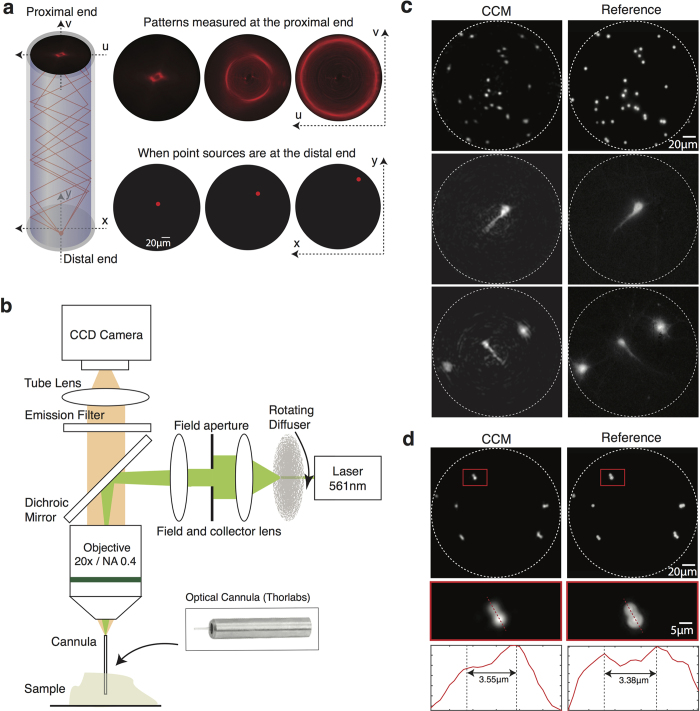
Computational-Cannula Microscopy (CCM) in the epi configuration. (**a**) Fluorescence from a point source enters the distal end of cannula and is guided via total internal reflections. A pattern is formed at the proximal end of the cannula, which is unique to the location of the point source, *i.e.*, the system exhibits space-variant point-spread functions (SV-PSFs). Three (experimentally measured) SV-PSFs are shown (top) along with the corresponding point source locations (bottom). (**b**) Schematic of epi-fluorescence CCM. Objective and tube lens are set to image the proximal end of cannula for transferring the image to the sensor. A conventional widefield microscope is placed underneath the sample (only for glass slides) to capture reference images. This is omitted to simplify the diagram. (**c**) CCM images of fluorescent beads (first row) and cultured DIV 16 hippocampal neurons (second and third row) on glass slides. Left: the reconstructed images, Right: reference images. White dashed line represents the field of view (diameter = 200 μm). (**d**) Closely spaced micro-beads for resolution test. Region inside the red square is magnified in the second row. The corresponding line-scans through the red dashed lines are shown in the bottom row.

In order to image deep inside tissue with a minimally invasive probe, such as a single cannula, the excitation laser and the fluorescence signal must traverse via the same cannula. This is analogous to an epi-configuration in fluorescence microscopy, where a single objective lens is used for both excitation and to collect the emission. The epi-configuration CCM is illustrated in Fig. 1(b). The excitation laser has a wavelength of 561 nm (Laser Quantum, gem 561, 250 mW). A series of lenses is used to image the laser onto the proximal end of the cannula. A rotating diffuser is used to homogenize the excitation intensity in the distal end of the cannula. Details of the analysis of the uniformity of excitation over the entire field of view and design of the excitation system are included in the Supplementary information. Note that in the simplest configuration, an image sensor can be placed in close vicinity of the proximal end of the cannula. In the current implementation, an objective and a tube lens are used to transfer the image at the proximal end of the cannula to the sensor plane. An emission filter blocks any excitation light in the signal path.

In order to obtain the SV-PSFs for the system, we first placed a slide with one fluorescent bead (Invitrogen FluoSphere^®^ Sulfate Microsphere, 4 μm, 580/605) next to the distal end of the cannula. The slide was then stepped using a stage in a raster fashion across the entire field of view (step-size = 2 μm). At each step, the image in the proximal face of the cannula was recorded. Details of the sample preparation and the calibration process are described in the Supplementary information. Next, several imaging tests were performed using slides containing fluorescent beads spread across the field of view. Once the image on the proximal end of the cannula was recorded, the reconstruction algorithm described earlier was applied. An example of the reconstructed image (left) and the reference image (right) are shown in Fig. 1(c). Next, we used fixed cultured hippocampal neurons transfected with a red fluorescent protein, td-Tomato, to ascertain whether the cannula can resolve biological samples. The reference image was obtained using a conventional fluorescence microscope that was built on the other side of the sample (not shown). CCM in the epi-configuration is able to successfully reconstruct the image of the fluorescent beads and neurons (Fig. 1).

The numerical aperture of our imaging system is constrained by the cannula, which has an NA of 0.39 and a diffraction-limited resolution of 0.77 μm (center wavelength = 0.6 μm). However, calibration parameters, most importantly, the calibration step size, which is 2 μm in this experiment, as well as noise introduced via the numerical-inversion process determine the practical resolution limit of the current implementation of CCM. Note that we have previously demonstrated 1 μm resolution, while imaging fluorescent beads on a glass slide in air, which was close to diffraction-limit of that system^17^. The theoretical resolution of our reference microscope (20x/NA = 0.5 objective) is about 0.6 μm (center wavelength = 0.6 μm). In Fig. 1(d), we demonstrate CCM images of a complex configuration of fluorescent microspheres and compare this against the reference image. In this image, we were able to resolve microspheres spaced by a distance of about 3.55 μm using CCM.

### Deep-tissue imaging of a phantom brain

**Figure 2:**
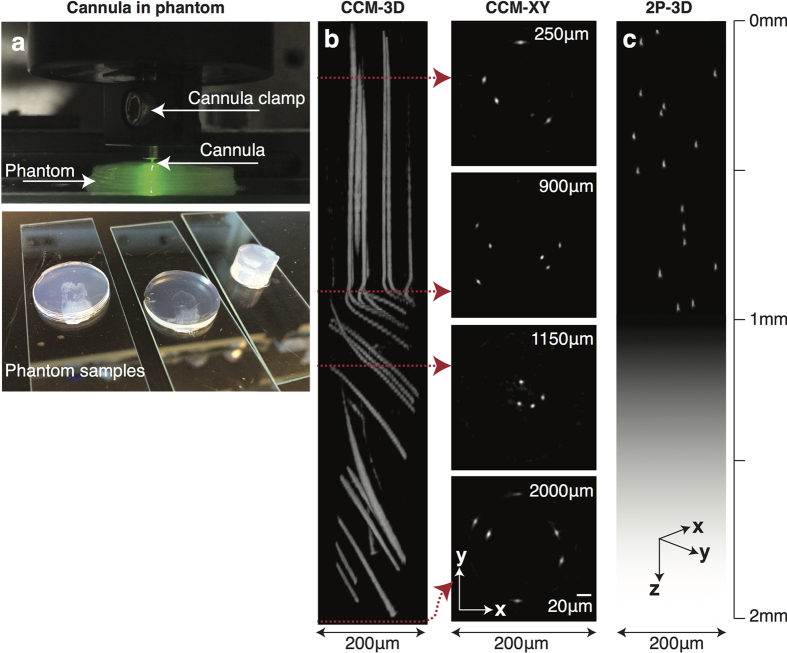
Deep-tissue imaging inside a phantom brain. (**a**) Photographs of phantom with embedded cannula (top) and phantom samples (bottom). (**b**) 3D rendering of CCM image stack and XY planes indicated by the red arrows (animation in Supplementary data). The cannula pushes the fluorescent beads forming the traces in the XZ image. Diagonal trace for Z > 900 um was due to lateral stress put on the phantom by cannula clamp. (**c**) Two-photon micrograph of the same phantom but at a different location than that in (**b**). No data is obtained for Z > 1 mm, since the working distance of the objective is ~1 mm.

Our first attempt at deep-tissue imaging was with a phantom tissue comprised of cross-linked agarose polymer (98% water) embedded with fluorescent micro-spheres. Agarose is well known to mimic the mechanical properties of brain tissue^25^. Hence, the agarose phantom tissue provides a simplified model to test the mechanical stability of the cannula during the implantation procedure. It is important to ensure that the cannula does not deform or distort during the implantation. Otherwise, the calibration data (SV-PSFs) will not be valid. Details of the preparation of the phantom samples are described in the Supplementary information. The cannula was carefully inserted into the phantom as illustrated in the photograph in Fig. 2a. The cannula was stepped in Z (into the sample) at a step size of 5 μm. At each step, the image at the proximal end of the cannula was recorded and reconstructed to verify valid image reconstruction during the insertion process. The CCM images are shown in Fig. 2b. The XZ projection indicates that the cannula pushed the fluorescent beads in its path in the Z direction resulting in continuous lines of fluorescence, as the cannula is expected to linearly compress the tissue that can lead to irreversible tissue damage. Therefore, *in vivo* application of the cannula probe will require insertion protocols developed for regular endoscopic imaging to mitigate tissue damage^26^ with the significant advantage that trauma is reduced due to the smaller cannula diameter. The maximum penetration depth of the cannula was 2 mm but in practice, the imaging depth is only limited by the length of the cannula. CCM images in the XY planes at various depths up to 2 mm are also shown. Because of our inability to perform registration, we were not able to obtain reference images from the same part of the phantom using a two-photon microscope. Nevertheless, we show in Fig. 2(c) a representative XZ image from the same phantom (different location than that in Fig. 2b) using a commercial 2-photon microscope (Bruker Ultima with Olympus 20x/NA1.0 and Coherent Chameleon II laser at 920 nm). In this case, the 2-photon microscope was unable to obtain any images for depths greater than about 1 mm due to the working distance of the objective. In real biological tissue, the penetration depth of 2-photon microscopy will also be limited by the scattering and absorption properties of the tissue.

### Deep-brain Imaging

A sample for deep-brain imaging was obtained by dissecting brain tissue at postnatal day 3 (P3) from transgenic mice that express td-Tomato in the Hoxb8 subpopulation of microglia in the brain^27^. We used Hoxb8^IRES-Cre/+^; CX3CR1^GFP/+^; Rosa26^CAG-LSL-tdTomato/+^ transgenic mice in which sub-population of Hoxb8 microglia was labeled with Rosa26^CAG-LSL-tdTomato/+^ reporter^28^. To ensure the integrity of the brain, we performed CCM within 30 minutes of the dissection. The sample was glued to the surface of a petri dish using instant glue (Krazy glue, all-purpose) and then immersed in phosphate-buffered saline (PBS) solution to prevent it from drying.

**Figure 3:**
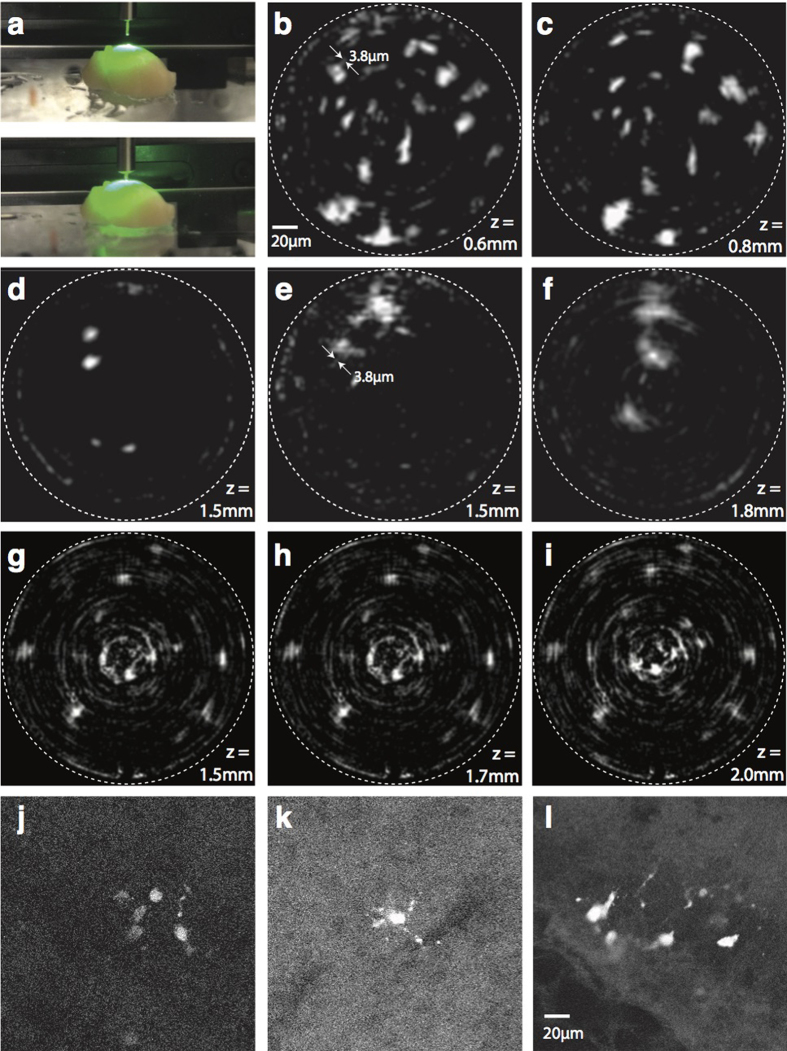
Deep-brain imaging of microglial cells inside the whole mouse brain using CCM. (**a**) Photograph of sample with embedded cannula. CCM images at 5 different locations and depths of (**b**) 0.6 mm, (**c**) 0.8 mm, (**d**) 1.5 mm, (**e**) 1.5 mm, and (**f**) 1.8 mm. (**g**) Images from a different sample (fixed mildly) at depths of (**g**)1.5 mm, (**h**)1.7 mm and (**i**)2 mm. The 3 images (**g**–**i**) are taken from the same XY location but at different depths. Arrows in (**b**) and (**e**) indicates measured width of cellular features showing ~3.8 μm, close to the measured resolution shown in Fig. 1(d). Two photon images of Hoxb8 microglia cells acquired from the same brain (**j**–**l**) images were taken. Similar morphology of cells is observed in images captured by both techniques. Animation of the 3D images are included in Supplementary data.

The cannula was carefully inserted into the brain near the frontal lobe at various locations, and the images at the proximal end of the cannula were recorded at various depths (Fig. 3a). Exemplary CCM images at depths of 0.6 mm, 0.8 mm, 1.5 mm, 1.5 mm and 1.8 mm at different locations within the brain are shown in Fig. 3(b)–(f), respectively. In the P3 brain, most microglial cells are immature and have few or no protrusions. Using CCM, we observed circular cell bodies with no protrusions, elongated cells without protrusions, and cells with a few short protrusions around their bodies. For qualitative comparison, two photon images were captured from the same P3 brain that CCM images were acquired. Most of the cells imaged using two photon microscopy show circular cell bodies, with a few cells occasionally showing short protrusions. As the depth increased, the two-photon image (Fig. 3k) becomes noticeably noisier due to an increase in the photomultiplier tube (PMT) gain in order to compensate signal loss due to light scattering and absorption. As the cannula was advanced carefully into the brain, we captured frames at each micro Z step, and reconstructed the CCM frames in post-processing. The resulting video of the fluorescent signal as well as additional images from other brain samples are included as Supplementary data. A series of images taken at the same XY location but at 3 different depths is shown in Fig. 3(g)–(i). Images shown in Fig. 3(g)–(i) shows observed axial translation of cells during cannula insertion.

## Discussion

In all brain-imaging methods, there is a trade-off between the depth, field of view, resolution, and potential for trauma. We summarize the most common brain-imaging methods based on these metrics in the Supplementary information (Table S2) and emphasize that CCM provides the optimal combination of these metrics. In general, two-photon microscopy has similar resolution to single-photon microscopy due to the requirement for long wavelength excitation. Resolution in CCM is constrained not only by the optical system but also by the noise introduced during the matrix-inversion process. This noise is not only dependent upon the system matrix of the cannula, but also on the details of the object. Analysis of the impact of label density and noise on the performance of CCM has been reported recently^29^. Although we have also demonstrated imaging of dense fluorescent beads (see Figure S7 in Supplementary information), it is clear that CCM works particularly well for sparsely labeled samples. Although more work is required to demonstrate this, one potential application of CCM is in deep-brain calcium imaging with low density of expression. Finally, we emphasize that CCM is able to minimize tissue damage for deep-brain imaging due to its small size, while attaining sufficient resolution, field of view and the benefit of fast widefield image acquisition. Furthermore, CCM is elegantly simple, since it only requires a cannula and computation.

We further note that the cannula used in CCM is the same as that used exhaustively in optogenetics. As a result, we can expect full compatibility of CCM with existing protocols for deep-brain optogenetics. Furthermore, since the cannula is made of glass, no structural deformation is expected during imaging or implantation, as confirmed by sustained imaging capability during probe insertion with the presence of mechanical pressure at the tip of the probe.

## Conclusion

Imaging deep inside tissue, including the brain, is critical to understanding various biological processes. Doing so through a small probe is also of primary importance for minimizing tissue damage. Here, we apply computational techniques to create fluorescent images and video using a microscopic surgical needle (cannula) to guide light in and out of a mouse brain. The simplicity and small footprint of our system have the potential for deep-brain imaging (depths > 1 mm) which should enable a wide variety of biological and neuroscience studies in the future. Although all our images were performed on dissected brains, the technique can be extended with slight modifications to awake behaving animals^30^.

## Methods

### Fluorescent microbead sample preparation

For both calibration and reconstruction validity check, we used microbead samples of various densities. The microbead of choice was 4 μm red FluoSpheres sulfate microsphere (580/605). Spectrum of the chosen microbead is shown in the Supplementary Figure 4. To make samples with desired density, diluted the bead solution (2% solid) to 1:500 high purity water, then vortex the diluted solution for 2 minutes. The 1:500 solution is diluted once more to 1:10 high purity water to make 1:5,000 solution, followed by vortex. Repeat the same dilution process to the to create 1:50,000 solution. To make a calibration slide, aliquot 50 uL of 1:50,000 diluted solution onto a glass slide. Aspirate with pipette and let it completely dry. Note that we do not use coverslip to locate the calibration bead right in front of the distal end of cannula. Once prepared, we inspect the sample is well prepared under the reference microscope. Calibration slide is used for calibration process, which is described in the next section of the Supplementary information. To make an imaging slide, aliquot 50 μL of either 1:500 or 1:5,000 diluted solution onto a glass slide. Aspirate and completely dry. After calibrating the CCM, we use this imaging slide to test proper operation of the system. See Supplementary Figure 5 for sample data taken from those imaging slides.

### Acquiring and processing calibration images

For calibration, a single 4 μm microbead is located and scanned across the field while its projected cannula pattern is captured at every 2 μm. Once scanning is complete, images are imported into Matlab and collated into one variable. For each image, we perform pipeline of image processing to ensure linearity between every image collected. Images of multiple exposures are combined into one using an HDR (high dynamic range) algorithm, and then background pattern is subtracted, and lastly we mask unnecessary part of the image out. Then, we perform singular value decomposition (SVD) onto the calibration matrix. SVD decomposes matrix into two orthonormal bases, **U** and **V**, and singular vector **s**, all of which are later used by Tikhonov regularization to reconstruct CCM images.

### Cultured neuron sample preparation

Hippocampal neuron cultures were prepared from E18 rat embryos. Hippocampi were dissected out of the brain and dissociated in 0.67 mg/mL papain (Worthington)/0.01% DNase (Sigma-Aldrich) solution for 20 min in a water bath at 37 °C. The tissue was then triturated in NM5 media (5% horse serum, 2% GlutaMAX, 1% penicillin/streptomycin, 2% B-27 supplement, Gibco) with fire-polished glass Pasteur pipets. The cell suspension was filtered through a 70-μm filter, centrifuged for 4 min at 800*xg*, and the pellet re-suspended in fresh NM5 media. Neurons were plated on No. 1 glass coverslips in 12-well tissue culture dishes at 9 × 10^4^/mL. On DIV4, neurons were fed in a half-media exchange with glia-conditioned NM1 (1% horse serum, 1% GlutaMAX, 1% penicillin/streptomycin, 2% B-27 supplement) and AraC (Sigma-Aldrich) to stop glia proliferation, and fed every 3 days thereafter with glia-conditioned NM1 without AraC. On DIV8, neurons were transfected with tdTomato using Lipofectamine 2000 (Thermo Fisher Scientific). 16 hr later, neurons were fixed in 4% paraformaldehyde/4% sucrose and the glass coverslips were mounted neuron side down in Fluoromount (Sigma-Aldrich) on a glass slide. More CCM reconstruction of neuron samples can be found in Supplementary Figure 8.

### Agarose phantom preparation

Phantom used for experiment (Fig. 2) was made in 2% agarose gel, prepared via the following steps. First, measure 0.4 g of agarose powder (Sigma-Aldrich A9311). Second, add agarose powder and 20 mL of high purity water to a flask and stir it until agarose disperses uniformly in the solution. Place the flask in a microwave oven and heat using 100% power for 10 seconds. Repeat 10 seconds interval heating until agarose completely dissolves. Gently stir between intervals to suspend agarose. Before agarose solidify, add 100 μL of 1:500 diluted bead mixture (SI section 2) into the agarose. Mix the solution well using vortex. Place a mold on a glass slide, and pour generous amount of agarose solution to fill the mold. Then, wait few minutes until agarose solidify. Glue the solid agarose gel to the glass slide to prevent sample from sliding. Additional data taken from the agarose phantom can be found in Supplementary Figure 9.

### Brain dissection and sample preparation

Postnatal day 3 mouse pups from Hoxb8^IRES-Cre/+^; CX3CR1^GFP/+^; Rosa26^CAG-LSL-tdTomato/+^ mice were carefully dissected following isoflurane anesthesia. Brain tissues were kept in 1X phosphate buffered saline solution (PBS) and subsequently used for imaging. For fixed preparation, brains from the CX3CR1-GFP/+Hoxb8-IRES-Cre/+RosaTdTomato/+ mice were first perfused with 1X PBS followed by 0.1% paraformaldehyde. Tissues were preserved overnight in 0.1% paraformaldehyde solution, washed 3 times with 1X PBS and soaked in 10%, 20 and 30% sucrose solutions on day 1, 2 and 3. From day 3, the tissues were used for imaging. More data taken from these brains can be found in Supplementary information (Supplementary Figures 10–12).

### Ethics Statement

All procedures were performed in accordance with the guidelines of the institutional animal care committee of the University of Utah, and in conjunction with NIH guidelines.

All procedures were approved by the institutional animal care committee of the University of Utah, and in conjunction with NIH guidelines.

## Additional Information

**How to cite this article**: Kim, G. *et al*. Deep-brain imaging via epi-fluorescence Computational Cannula Microscopy. *Sci. Rep.* **7**, 44791; doi: 10.1038/srep44791 (2017).

**Publisher's note:** Springer Nature remains neutral with regard to jurisdictional claims in published maps and institutional affiliations.

## Supplementary Material

Supplementary Video 1

Supplementary Video 2

Supplementary Video 3

Supplementary Video 4

Supplementary Video 5

Supplementary Video 6

Supplementary Video 7

Supplementary Information
