# Supplementary material for: Deep-brain imaging via epi-fluorescence Computational Cannula Microscopy
Source: Sci Rep. 2017 Mar 20;7:44791. doi: 10.1038/srep44791 (PMC5357895; doi:10.1038/srep44791)
Supplement: Supplementary Information [file srep44791-s8.pdf]

Supplementary Information for
Deep-brain imaging via epi-fluorescence Computational Cannula Microscopy

Ganghun Kim,[1] Naveen Nagarajan,[2] Elissa Pastuzyn,[3] Kyle Jenks,[3] Mario Capecchi,[2] Jason Shepherd,[3] and Rajesh Menon[1,*]
[1]Department of Electrical and Computer Engineering, University of Utah, Salt Lake City, UT 84112
[2]Department of Human Genetics, University of Utah, Salt Lake City, UT 84112
[3]Department of Neurobiology and Anatomy, University of Utah, Salt Lake City, UT 84112

[*] rmenon@eng.utah.edu

1. Optical setup

Detailed diagram of our optical setup is explained here. Following is the complete schematic of the system, parts list table, and its photograph.

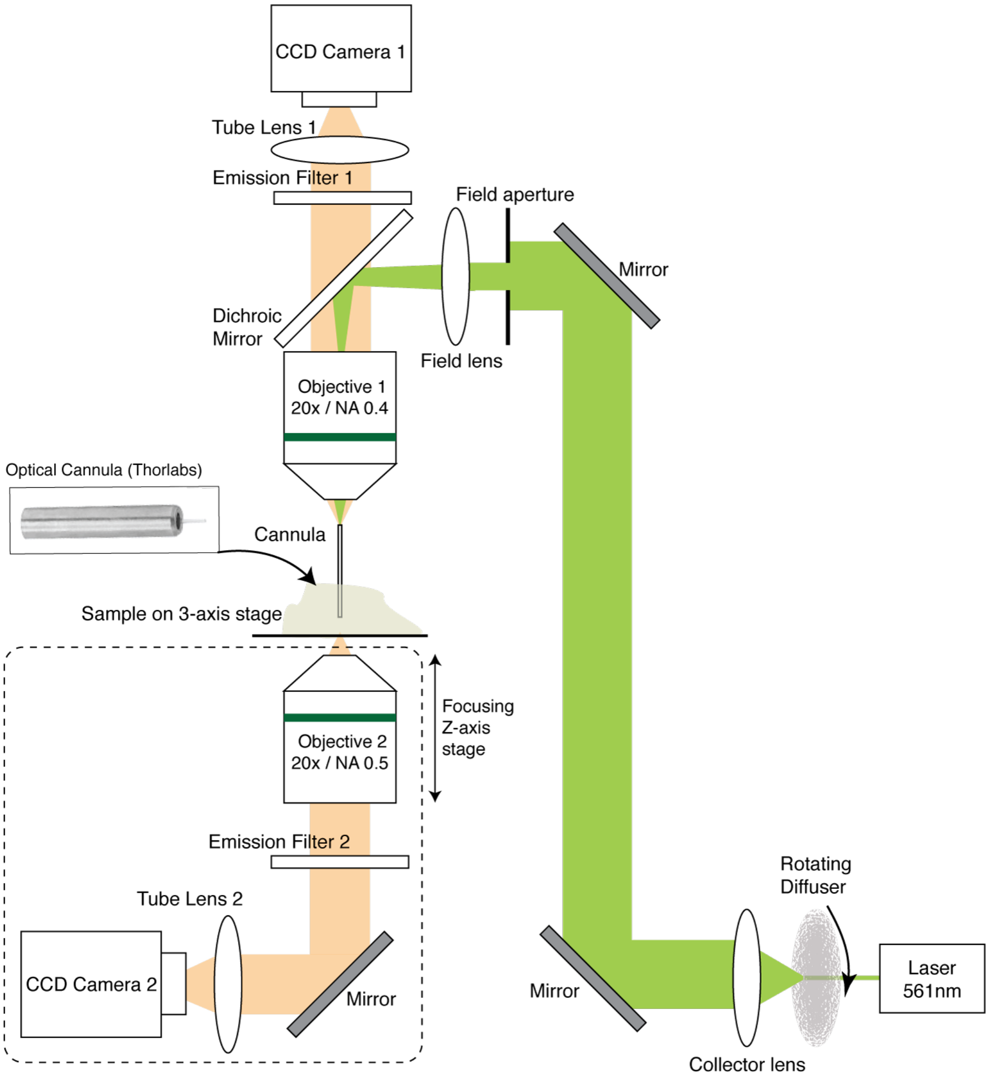

Figure S.1. Comprehensive schematic of optical setup used for the experiment. Inside dashed line is the reference microscope used for calibration and fluorescent bead imaging. It is mounted on a translational stage that moves along z-axis for focusing.

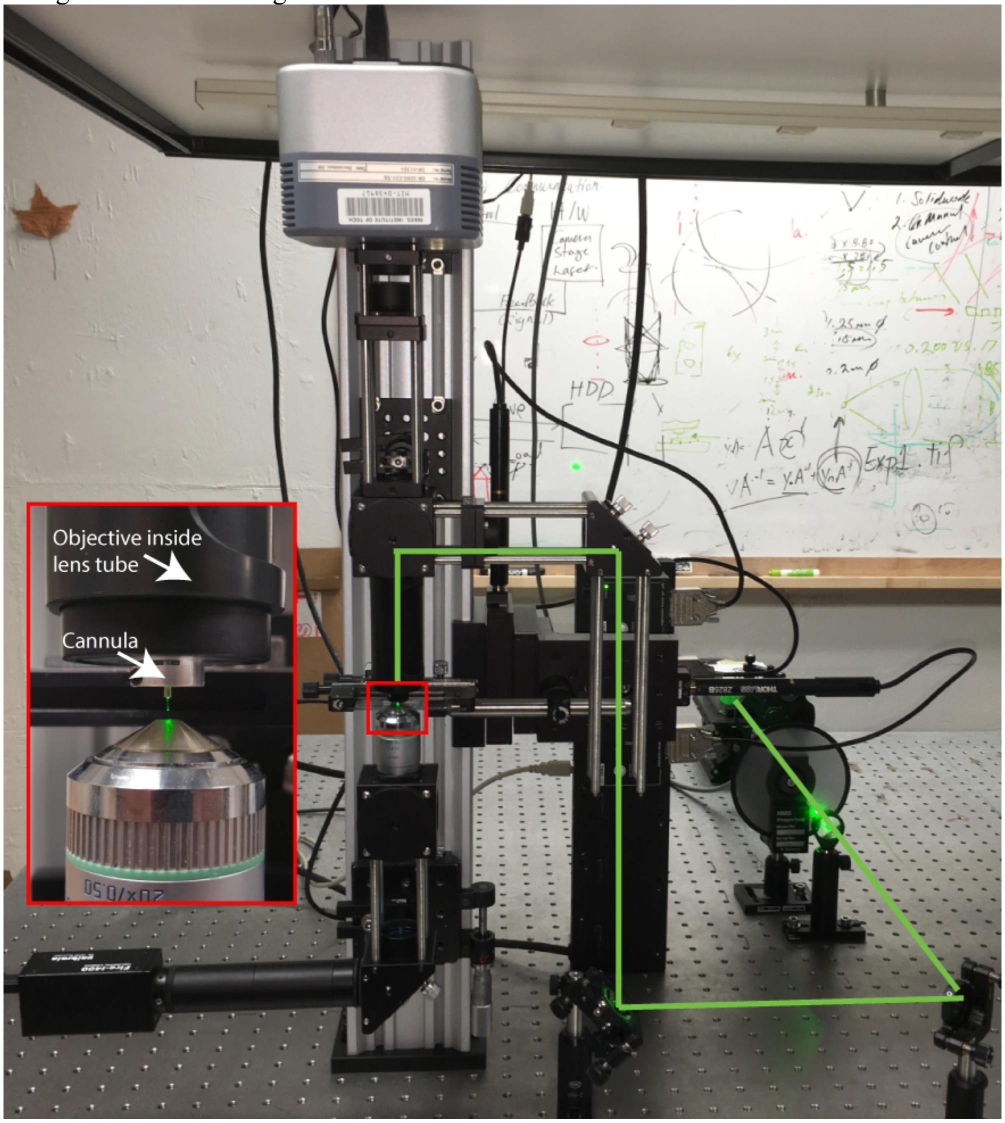

Figure S.2. Photograph of the optical setup. Green line shows the excitation laser path. Note the CCM objective is placed inside the lens tube right behind the cannula, hence not visible in the picture. Close-up view inside red square shows the arrangement of the cannula probe and reference microscope objective.

	**Name**	**Part**
**Computational cannula microscope**	Cannula	Thorlabs CFMC12L02
	Objective 1	Newport M-20X, 20x/NA 0.4
	Emission filter 1	Thorlabs FEL600
	CCD Camera 1	Andor Clara Interline CCD
	Dichroic mirror	Thorlabs MD568
	XYZ Stage	Thorlabs PT3-Z8
	Tube lens 1	Thorlabs LA1131-A, f=50mm
**Reference**	Objective 2	Leica HC PL FLUOSTAR 20x/NA 0.5

**microscope**	CCD Camera 2	Unibrain Firewire-400
	Emission filter 2	Thorlabs FEL600
	Tube lens 2	LA1986-A, f = 125mm
**Quasi-incoherent Illumination**	Laser	Laser Quantum Gem 562nm, 250mW
	Collector lens	Thorlabs LA1131-A, f=50mm
	Field lens	LA1509-A, f=100mm
	Rotating Diffuser	HMS light beam chopper 220 with diffuser film

Table S.1. Parts used to construct the computational cannula microscope, reference microscope, and quasi-incoherent illumination source used for fluorescent excitation.

Conventional microscopes use Kohler illumination to achieve homogeneous illumination pattern. We adopted similar strategy to achieve uniform illumination in our computational cannula microscope (CCM). First, laser beam passes through a rotating diffuser to create a quasi-incoherent (temporally coherent, but spatially incoherent) illumination. We needed incoherent illumination source because coherent beam produces unwanted speckle patterns at the distal end of cannula due to interference (Fig. S.3). For the future implementation, LED sources could be used to eliminate the need of diffuser.

Then, we treated the diffuser surface as if it is a new incoherent light source, and formed a Kohler illumination of the new source using set of lenses. Collector lens collimate the light scattered off the diffuser surface, which is then focused to the back focal plane of the CCM objective via the field lens. Hence, we achieve homogeneous illumination pattern at the proximal end of the cannula. We found that the illumination contains broad angular spectrum and uniform spatial distribution results to a uniform pattern at the distal end of cannula.

We believe that the importance of such uniform illumination is greater than regular microscopy. When calibrating, non-uniform illumination lead to non-uniform signal-to-noise ration among different calibration images at different point source locations. When reconstructing, imbalance in calibration SNR could induce undesired artifacts and noise in the final reconstruction images.

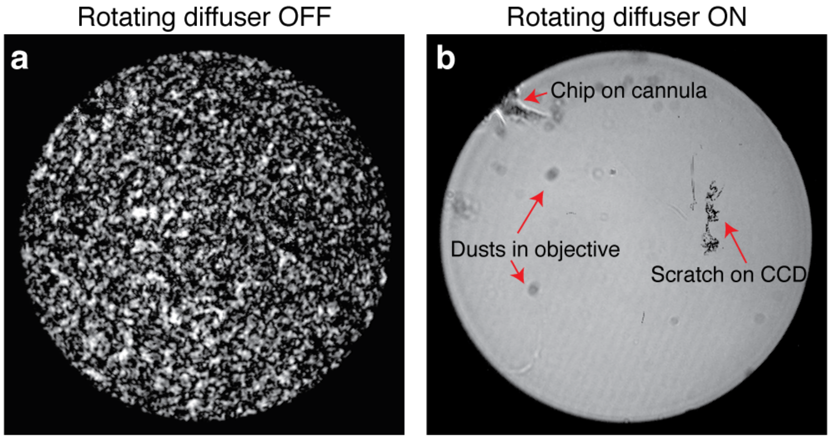

Figure S.3. Images of the excitation pattern are captured by the reference microscope without emission filter when (a) diffuser does not rotate and (b) diffuser is rotating. Dark spots around the top-left corner were caused by dust particles in the objective. Excitation doesn't appear very clean due to imperfections on the reference microscope itself, such as dust particles inside objective and scratch on the sensor array. Besides, excitation pattern appears uniform. A little chip damage is observed on the top-left corner of the image (b). However, damage was very minor and didn't cause artifacts in CCM imaging.

2. Acquiring and processing calibration images

For calibration, a single 4μm microbead is located and scanned across the field while its projected cannula pattern is captured at every 2μm. Once scanning is complete, images are imported into Matlab and collated into one variable. For each image, we perform pipeline of image processing to ensure linearity between every images collected. Images of multiple exposures are combined into one using HDR (high dynamic range) algorithm [1], and then background pattern is subtracted, and lastly we mask unnecessary part of the image out. Then, we perform singular value decomposition (SVD) onto the calibration matrix [2]. SVD decompose matrix into two orthonormal bases, **U** and **V**, and singular vector **s**, all of which are later used by Tikhonov regularization to reconstruct CCM images.

3. FluoSpheres® microbead sample preparation and its spectrum

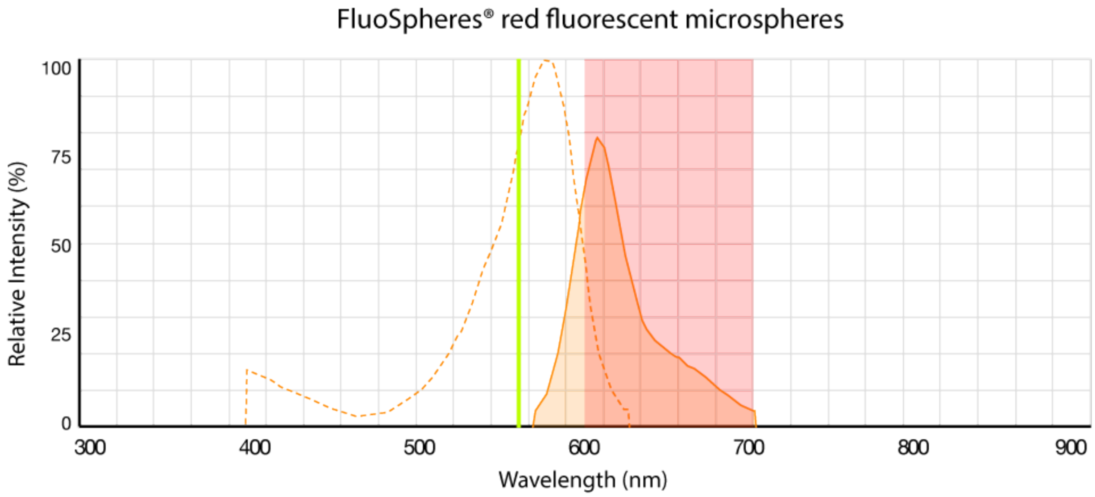

Figure S.4. Fluorescent excitation (dashed orange line) and emission (solid orange line) spectrum of red dye that coats 4μm FluoSpheres® microsphere used for experiment. Green vertical line indicates the excitation wavelength. Emission filter bandwidth (red box) is overlaid on the emission graph.

For both calibration and reconstruction validity check, we used microbead samples of various densities. The microbead of choice was 4μm red FluoSpheres® sulfate microsphere (580/605). Spectrum of the chosen microbead is shown in the supplementary figure 4. To make samples with desired density, diluted the bead solution (2% solid) to 1:500 high purity water, then vortex the diluted solution for 2 minutes. The 1:500 solution is diluted once more to 1:10 high purity water to make 1:5,000 solution, followed by vortex. Repeat the same dilution process to the to create 1:50,000 solution. To make a calibration slide, aliquot 50uL of 1:50,000 diluted solution onto a glass slide. Aspirate with pipette and let it completely dry. Note that we do not use coverslip to locate the calibration bead right in front of the distal end of cannula. Once prepared, we inspect the sample is well prepared under the reference microscope. Calibration slide is used for calibration process, which is described in the next section of the supplementary information. To make an imaging slide, aliquot 50uL of either 1:500 or 1:5,000 diluted solution onto a glass slide. Aspirate and completely dry. After calibrating the CCM, we use this imaging

slide to test proper operation of the system. Supplementary figure 5 six sample data taken from those imaging slides.

4. Cultured neuron sample preparation

Hippocampal neuron cultures were prepared from E18 rat embryos. Hippocampi were dissected out of the brain and dissociated in 0.67 mg/mL papain (Worthington)/0.01% DNase (Sigma-Aldrich) solution for 20 min in a water bath at 37°C. The tissue was then triturated in NM5 media (5% horse serum, 2% GlutaMAX, 1% penicillin/streptomycin, 2% B-27 supplement, Gibco) with fire-polished glass Pasteur pipets. The cell suspension was filtered through a 70-µm filter, centrifuged for 4 min at 800*xg*, and the pellet resuspended in fresh NM5 media. Neurons were plated on No. 1 glass coverslips in 12-well tissue culture dishes at 9 x 10^4/mL. On DIV4, neurons were fed in a half-media exchange with glia-conditioned NM1 (1% horse serum, 1% GlutaMAX, 1% penicillin/streptomycin, 2% B-27 supplement) and AraC (Sigma-Aldrich) to stop glia proliferation, and fed every 3 days thereafter with glia-conditioned NM1 without AraC.

On DIV8, neurons were transfected with tdTomato using Lipofectamine 2000 (Thermo Fisher Scientific). 16 hr later, neurons were fixed in 4% paraformaldehyde/4% sucrose and the glass coverslips were mounted neuron side down in Fluoromount (Sigma-Aldrich) on a glass slide. More CCM reconstruction of neuron samples can be found in supplementary figure 8.

5. Agarose phantom preparation

Imaging phantom used for experiment (Fig. 2) was made in 2% agarose gel, prepared by the following steps. First, measure 0.4g of agarose powder (Sigma-Aldrich A9311). Second, add agarose powder and 20mL of high purity water to a flask and stir it until agarose disperses uniformly in the solution. Place the flask in a microwave oven and heat using 100% power for 10 seconds. Repeat 10 seconds interval heating until agarose completely dissolves. Gently stir between intervals to suspend agarose. Before agarose solidify, add 100uL of 1:500 diluted bead mixture (SI section 2) into the agarose. Mix the solution well using vortex. Place a mold on a glass slide, and pour generous amount of agarose solution to fill the mold. Then, wait few minutes until agarose solidify. Glue the solid agarose gel to the glass slide to prevent sample from sliding. Additional data taken from the agarose phantom can be found in supplementary figure 9.

6. Brain dissection and sample preparation

Postnatal day 3 mouse pups from CX3CR1-GFP/+Hoxb8-IRES-Cre/+RosaTdTomato/+ mice were carefully dissected following isoflurane anesthesia. Brain tissues were kept in 1X phosphate buffered saline solution (PBS) and subsequently used for imaging. For fixed preparation, brains from the CX3CR1-GFP/+Hoxb8-IRES-Cre/+RosaTdTomato/+ mice were first perfused with 1X PBS followed by 0.1% paraformaldehyde. Tissues were preserved overnight in 0.1% paraformaldehyde solution, washed 3 times with 1X PBS and soaked in 10%, 20% and 30% sucrose solutions on day 1, 2 and 3. From day 3, the tissues were used for imaging. More data taken from these brains can be found in supplementary information (Supplementary figure 10-12).

7. Correlation analysis of space-variant point-spread-function (SV-PSF)

As in any imaging systems, characteristics of the CCM heavily depend on the point-spread-function its optics create. With cannula, the process of multiple total-internal-reflection (TIR) is complex and hence requires study in and of itself to obtain comprehensive analytical and mathematical description of the its PSF. Therefore, we tried to gain insights of the underlying process by empirically inspecting how SV-PSF varies as a function of point source location. The most notable feature we observed in many of our SV-PSF images was a ring pattern. Although not exact, patterns created closely resemble a ring shape. Additionally, the ring pattern created by a point source had a radius similar to the distance between the center of cannula and the location of point source (Fig S.5).

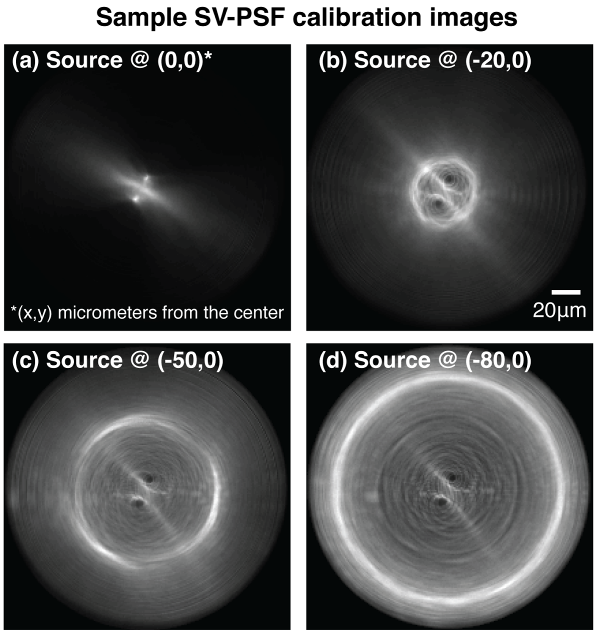

Figure S.5. Examples of space-variant point-spread-function (SV-PSF) when point source is at (a) the center, (b) -20µm, (c) -50µm, (d) and -80µm away from the center along the x axis. As point source moves away from the center, it creates a pattern similar to a ring whose radius is approximately the distance from the center of cannula to the point source.

As a result, SV-PSF created by a point source located along the perimeter of a circle are similar to each other, thought not exact. To quantify the effect, we measured Pearson correlation coefficient [3] between a selected sample PSF and every other PSF we collected for the calibration. Selected sample is a PSF image captured when point source was located at x = -50µm and y = 0um relative to the center (Fig S.5c). Following figure (Fig S.6) summarizes the analysis results. Analysis results indicate indeed a strong correlation between images along the same radial distance, 50µm for the particular sample we used. Also notice correlation drops much faster as a function of radius than it does along the polar angle, showing images located at different perimeter however are distinct from each other.

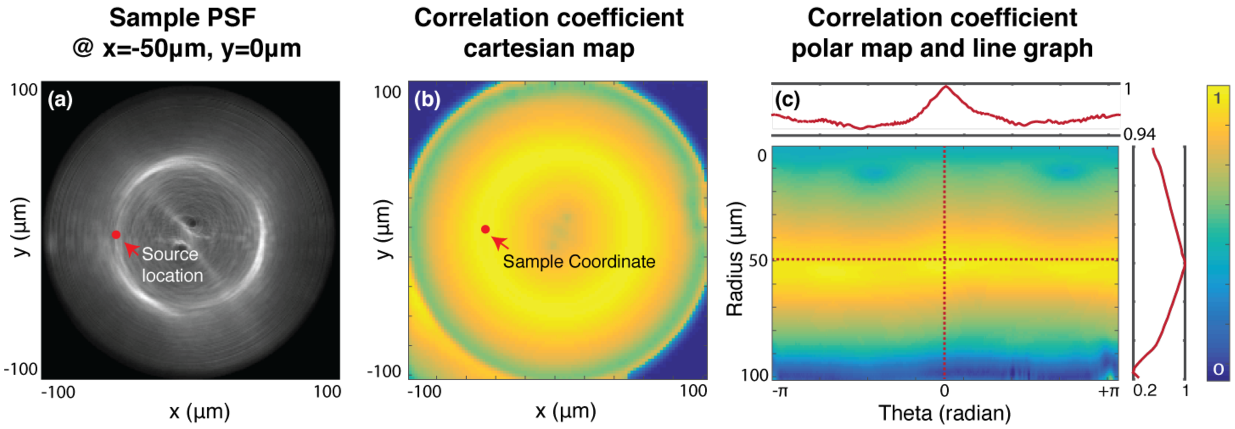

Figure S.6. Correlation analysis. (a) Selected sample PSF image from a point source located at -50μm from the center along the x-axis. (b) 2D Map of correlation coefficient between the sample image and all other SV-PSF images. (c) Same correlation coefficient data shown in polar coordinate. Cross section of the red dashed line is graphed along side. Note that the minimum value of the graph along theta is 0.94, while the minimum for the graph along radius is 0.2. Image (b) and (c) shares the same color bar.

In an ideal situation, it is best to have an orthogonal PSF matrix, whose correlation value is 0 everywhere except for with itself. However, we have shown with the number of example images that, despite of high correlation coefficient values, the slight variation between PSF images provide us with enough information to allow the reconstruction algorithm to pin point the exact location of fluorescent emission. We plan investigating further to reduce the effect of circular symmetry and lower correlation between calibration images in the future.

8. Supplementary fluorescent bead images

We used florescent bead samples to check performance of CCM. More images of 4μm fluorescent beads are provided along with corresponding cannula patterns. Each microsphere inside the field of view generates a slightly deformed ring, as described in the previous section. For example, three distinct ring pattern appears in the cannula pattern of Fig S.7a, which respectively corresponds to the bead located along its perimeter. Overall, we obtained images that accurately resemble conventional microscope images, confirming good performance of CCM system used.

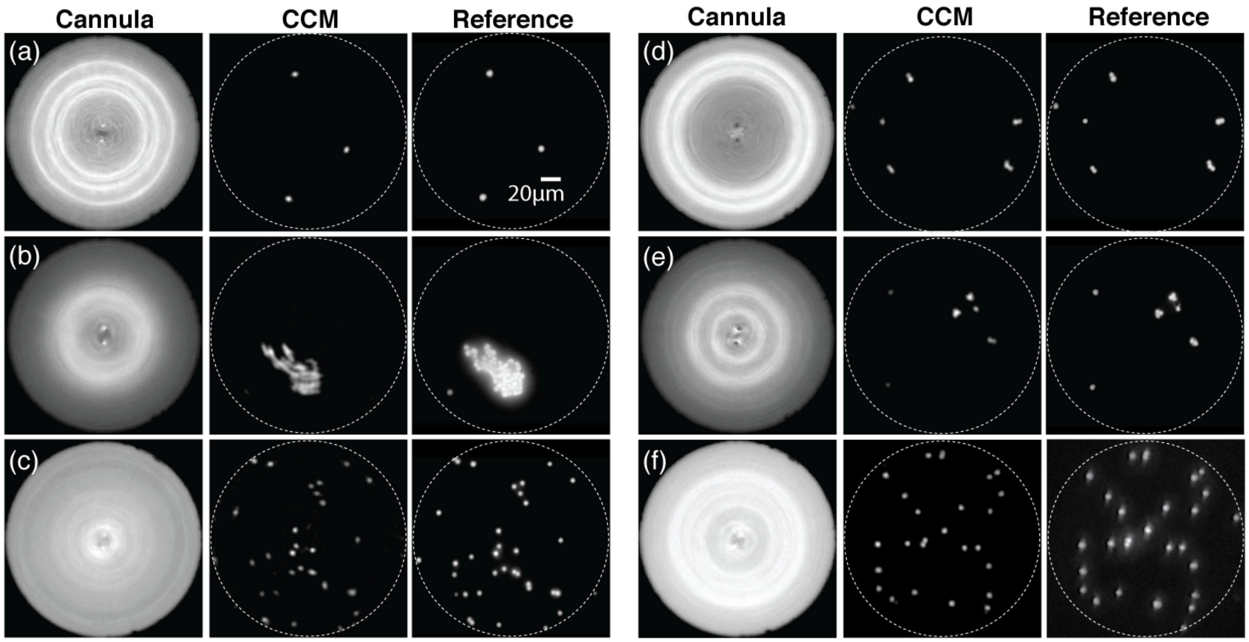

Figure S.7. Supplementary bead images. In each sample, first column shows the measured light intensity formed on the proximal end of cannula, second column shows its CCM reconstruction, and third column shows the image captured by the reference microscope. Image (c) and (d) was demonstrated in the main text as an example without their cannula patterns.

9. Supplementary cultured neuron images

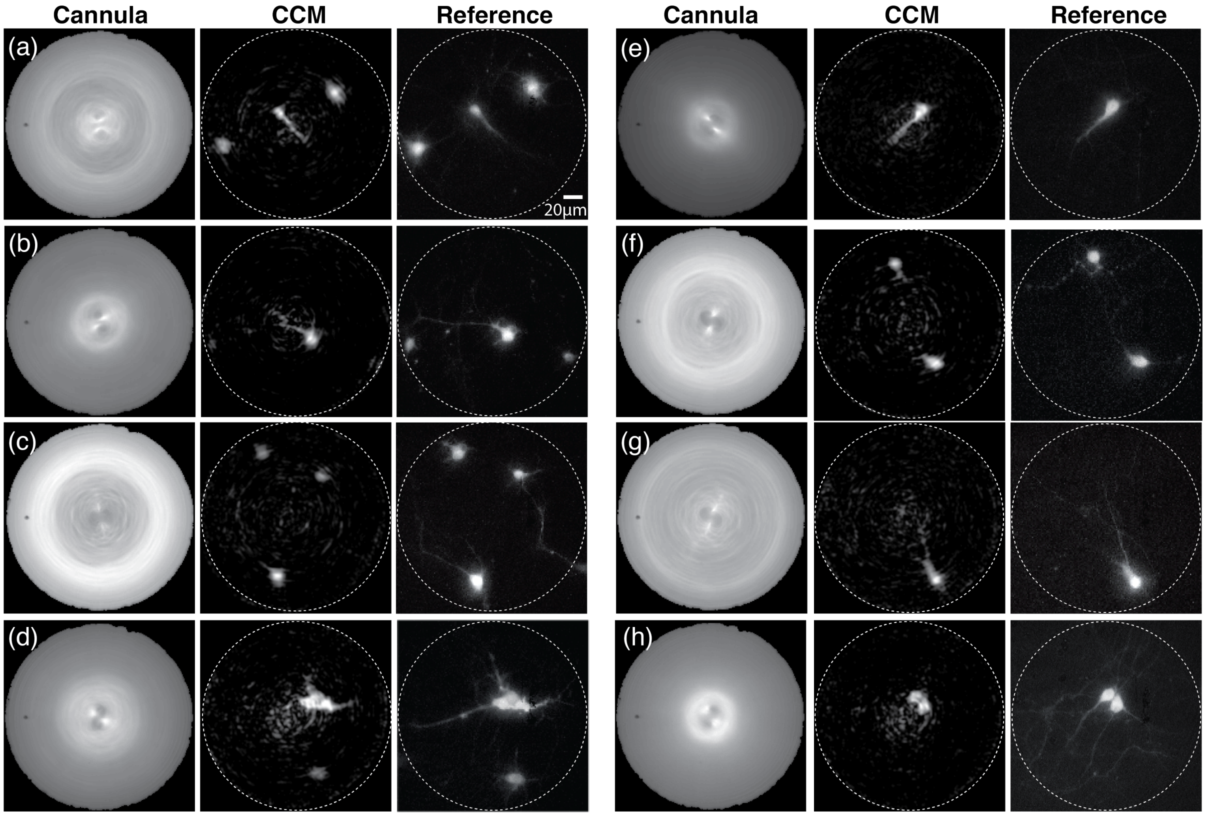

Figure S.8. Supplementary neuron images. Circular dash line indicates field of view. (a) and (e) was shown in the main text as primary examples without their cannula patterns.

In all images above, CCM could successfully reconstruct major structures of the neuron, including the cell body. Bright part of the protrusion, close to cell body, were also correctly imaged as signified in the image Fig S.8a. However, it struggled to recover all the fine synaptic features located father from the body. We believe that the large intensity difference between the cell body and protrusions are making the synaptic features difficult to reconstruct.

10. More XY image planes from the imaging phantom and its cannula pattern

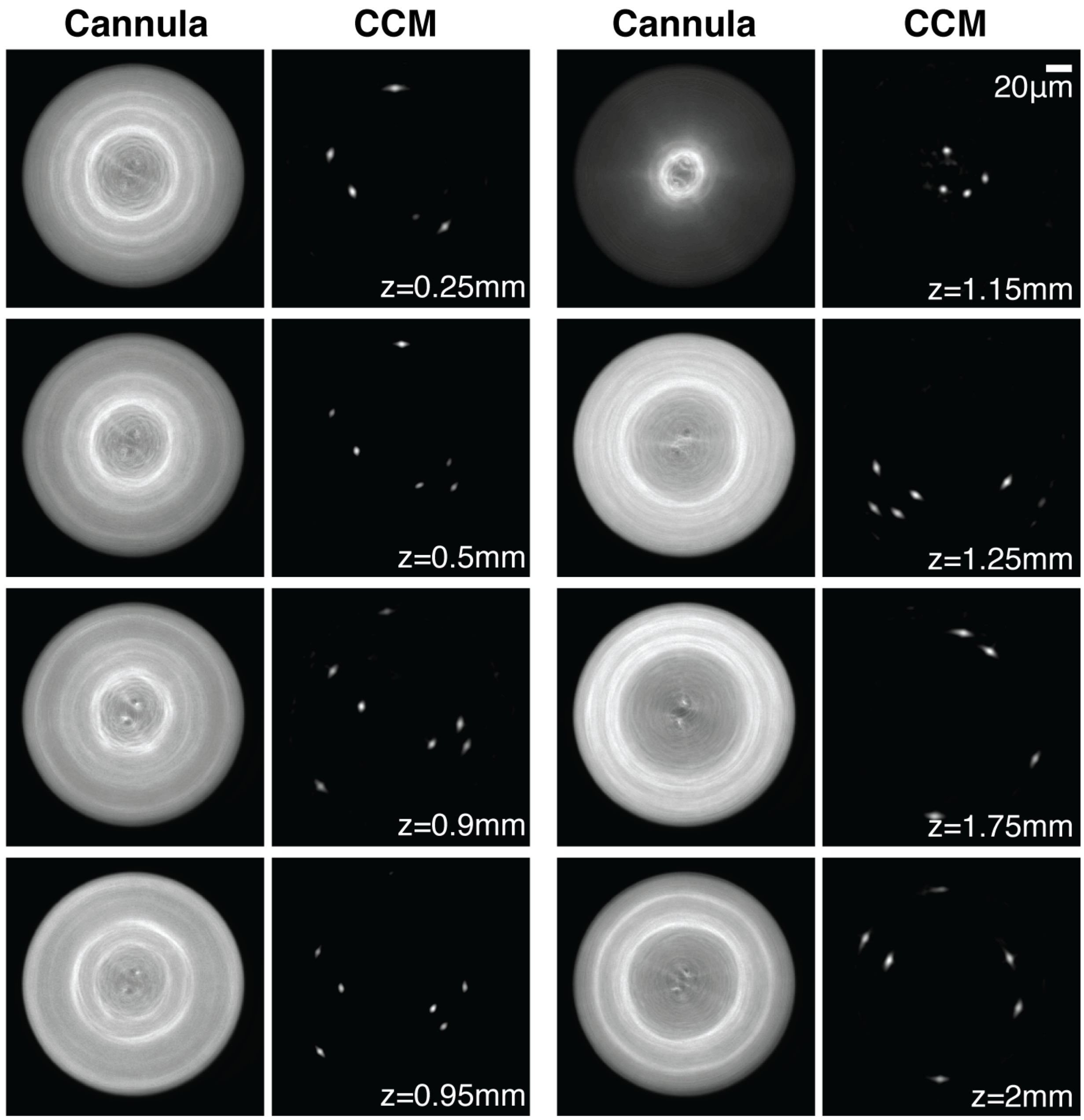

Figure S.9. Additional XY plane images from the z-scan stack, and their cannula patterns.

11. More CCM images from the brain

Additional images taken from the whole brain specimen are presented below. Each figure shows collection of selected images from the same brain sample, measured at various location and depth.

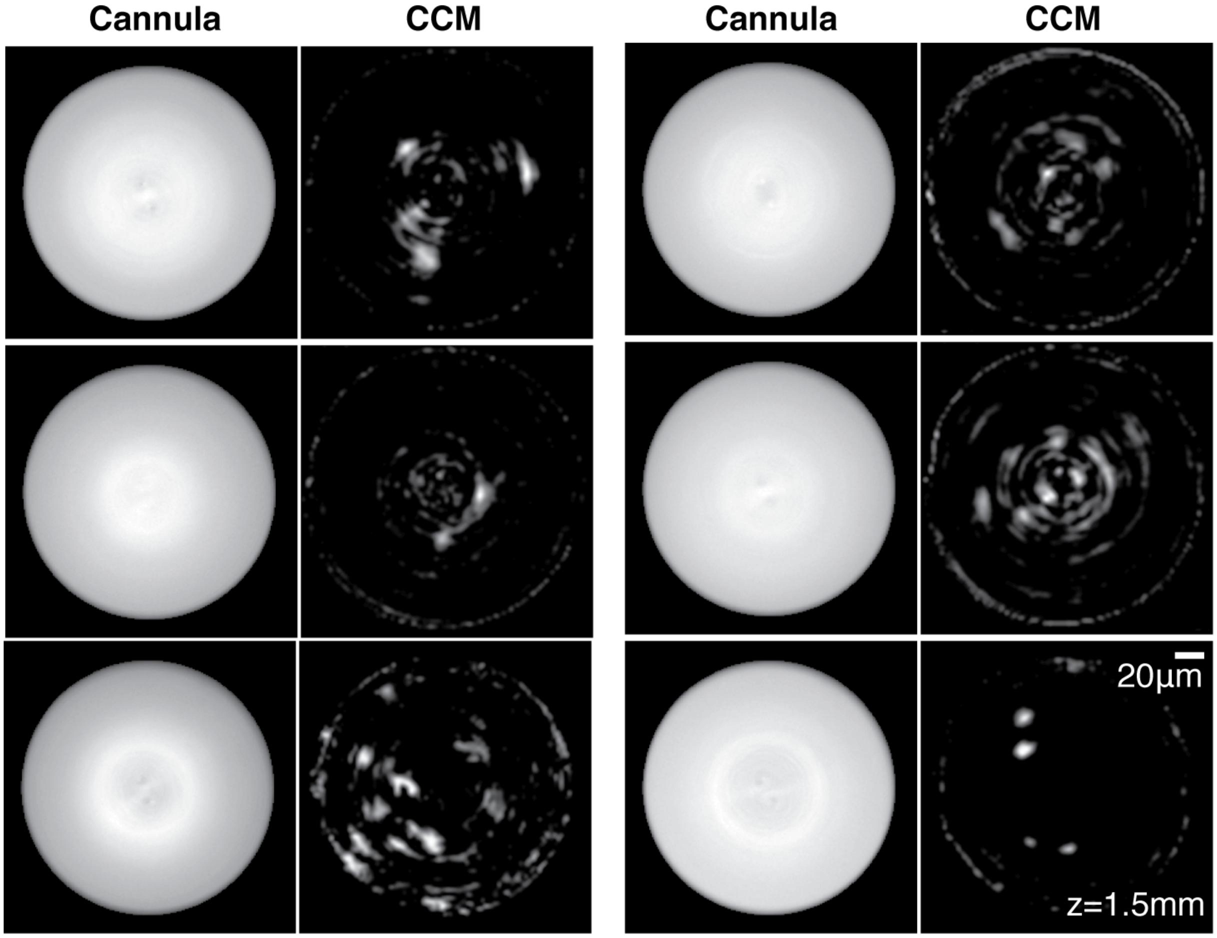

Figure S.10. Supplementary CCM reconstructions and their cannula measurements from a brain sample #1. Sample #1 was dissected from P0 post-natal stage mouse. No penetration information was recorded for this first experiment, except for the last data shown at the bottom-right.

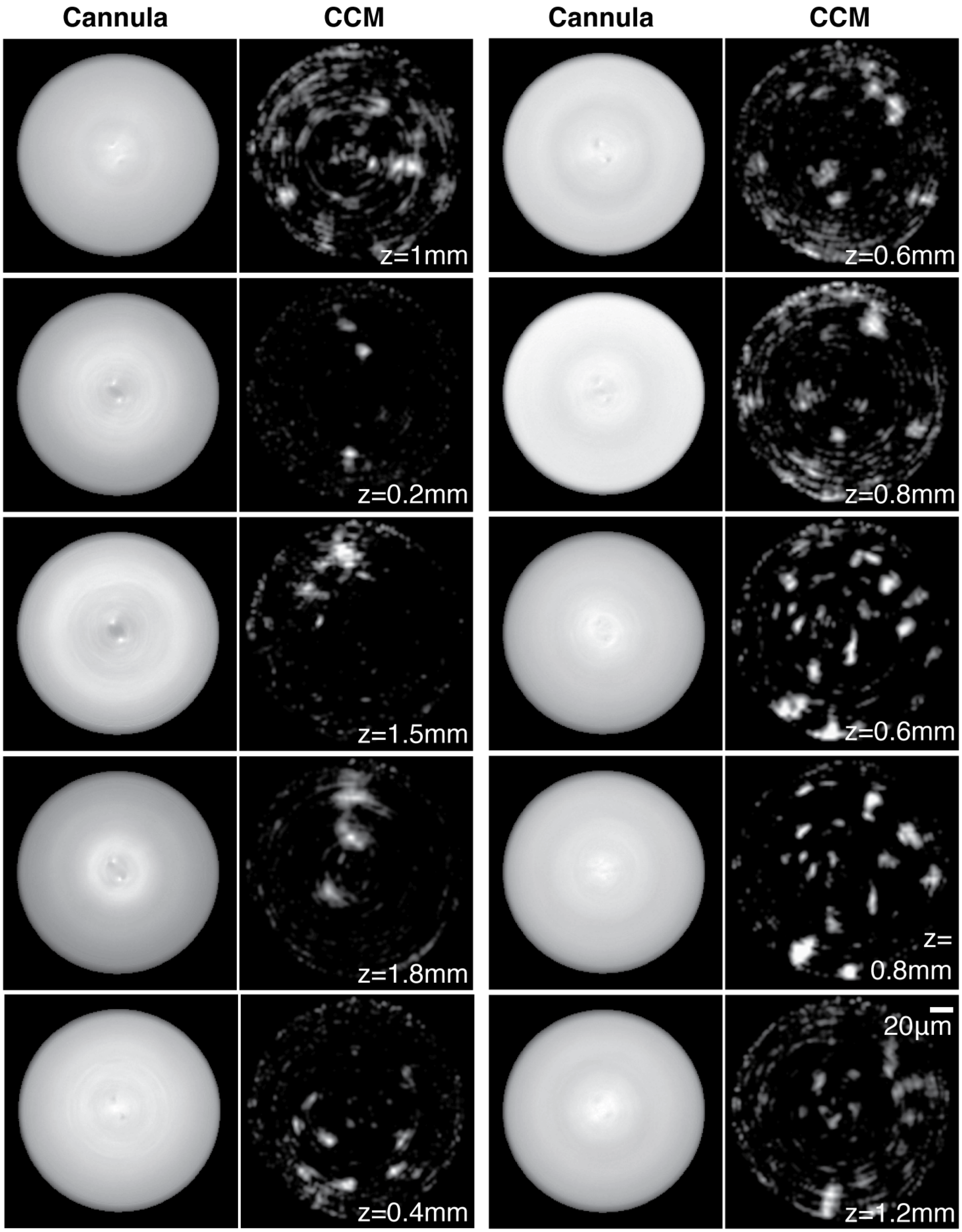

Figure S.11. Supplementary CCM reconstructions and their cannula measurements from a brain sample #2. Sample #2 was dissected from P3 post-natal stage mouse and imaged immediately after the dissection.

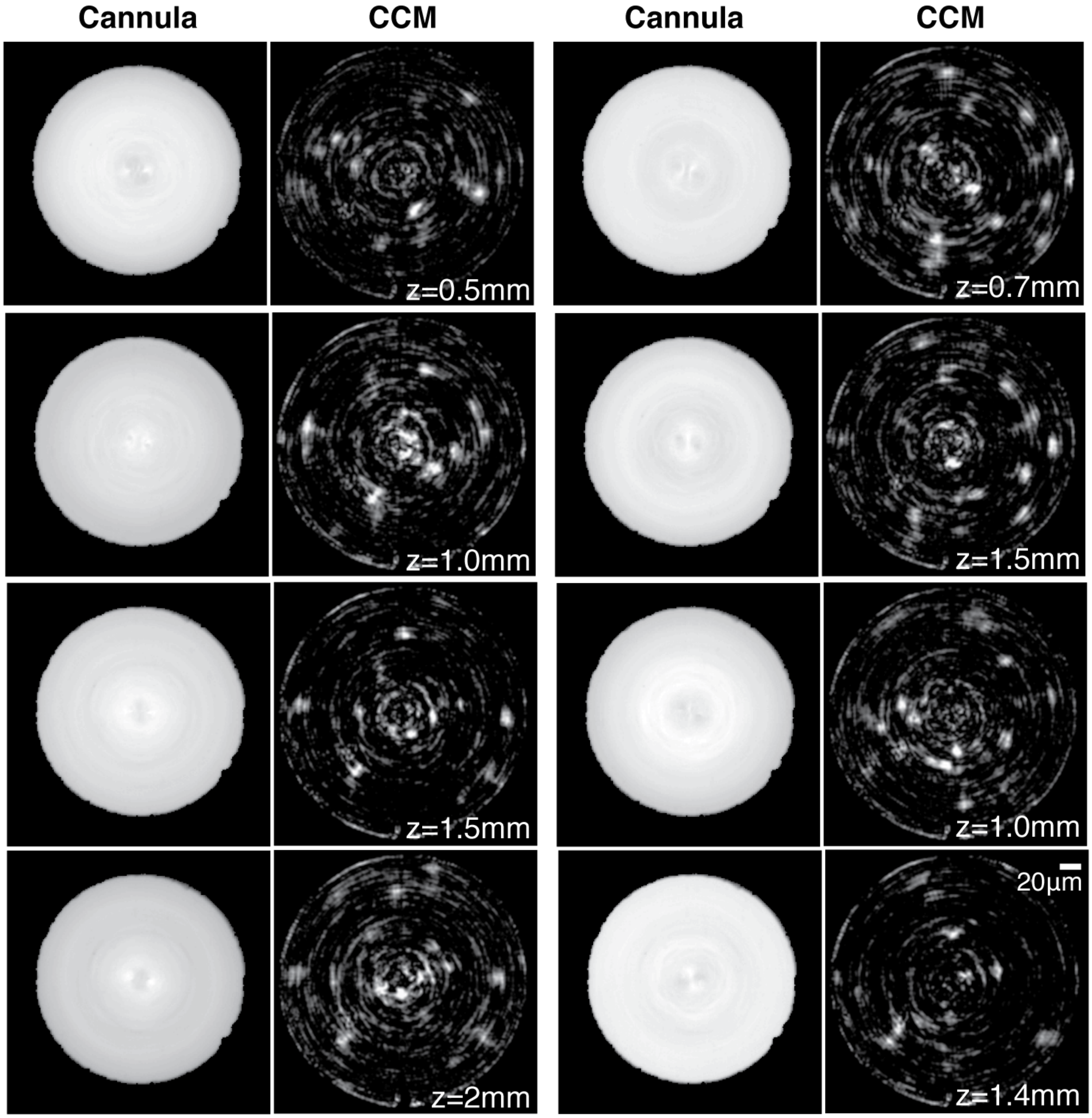

Figure S.12. Supplementary CCM reconstructions and their cannula measurements from a brain sample #3. Sample #3 was dissected from P3 post-natal stage mouse and fixed in a 0.5% PFA.

12. More two photon images from the brain

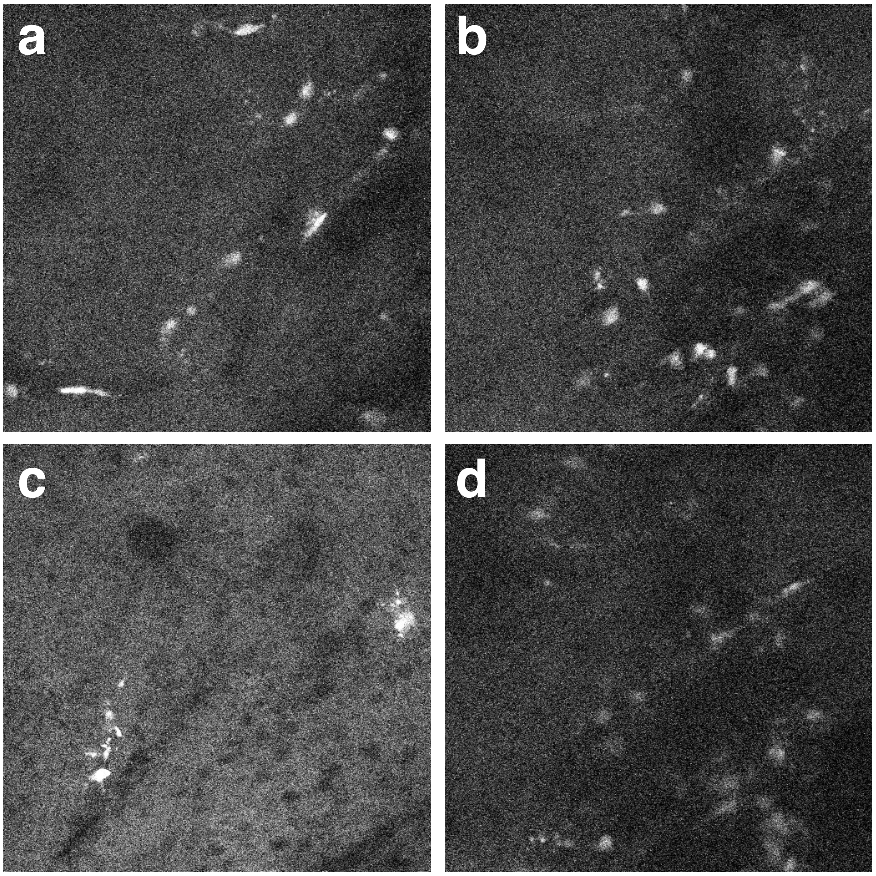

Figure S. 13. Supplementary two photon images obtained from the brain sample #3. Note the same brain is used to obtain CCM images presented in the figure 3.(g)-(i) as well as the supplementary figure 12 after the two photon imaging.

13. Description of the inverse problem

When fluorescence is emitted from a target object, it enters the distal end of cannula and experiences multiple total internal reflections inside until it exits to the proximal end. As a result, a scrambled light distribution is formed at the proximal end. Though the spatial information of a fluorescent object might not be directly seen on these scrambled patterns, they contain enough information about the object to allow computational reconstruction of the spatial distribution of the original fluorescent object. The relationship between original object *f* and scrambled image *g* can be described as a Fredholm equation of the first kind as formulated below.

$$g(u,v) = \int\int \boldsymbol{K}(x,y,u,v) \cdot f\,(x,y)\,dx\,dy$$

This equation above establishes a linear relationship between the two function *f* and *g* through the kernel function **K** that describes the relationship between the two functions. In a conventional terminology, **K** is a space-variant point-spread-function (PSF) that describes two dimensional pattern $g(u,v)$ as a function of point source location (x,y). One can solve the equation for function *f* given the accurate knowledge of **K** and *g*, and hence be able to image through the cannula. The above equation presents the underlying nature of the problem, but it cannot be directly applied to yield numerical solutions using computers. In practice, we discretize all functions and take the form of a system of linear equations as follows.

$$\boldsymbol{K}f - g$$

We then solve the linear equation for unknown vector *f* given the known relationship matrix **K** and known measurement vector *g*. Naturally, it becomes necessary that we first discover what **K** precisely is. Because light interaction inside cannula is highly complex, obtaining analytical form of **K** is not practical. Hence, we construct kernel function by empirically measuring the scrambled pattern of a finite size point source (i.e. fluorescent bead). To obtain holistic information of **K**, fluorescent bead is scanned along the uniform gird (x_i, y_i) while scrambled images are taken at each location, where i = 1…N, and N is number of scanning samples in each dimension. We refer this whole process as calibration. Calibration also accounts for any mechanical defects or manufacturing error, providing a robust imaging capability. As a rule of thumb, we use radius of fluorescent bead as a step size of the scanning to meet Nyquist sampling criteria. As discussed later in detail, step size used for calibration determines the resolution of the CCM system, until the size gets smaller than the diffraction limit.

Once calibrated, *f* can be recovered by applying various method of solving an inverse problem, given the knowledge of **K** and *g*. The simplest and most naïve approach is to use a least square solution *f* that satisfies following.

$$\min_f \|\mathbf{K}f - g\|_2$$

However, this method is highly sensitive to any imperfection and noise of the data, and often fails when **K** is not an orthogonal matrix. Matrix analysis of **K** revealed that our matrix **K** is not orthogonal and ill-posed. Hence, we opt to Tikhonov regularization algorithm, a widely used

regularization method for solving ill-posed discrete inverse problems [4]. In brief description, Tikhonov regularization has an additional constraint term to the least square method that limits l_2-norm of the solution under a user controlled value α. Solution of Tikhonov regularization f satisfies following condition.

$$\min_f \|\boldsymbol{K}f - g\|_2 \ \text{ subject to } \|f\|_2 \leq \alpha$$

The value α is also called smoothing factor, as larger α value effectively increases the smoothness while suppressing noise of the solution. It also consumes very little computation resources compared to the iterative optimization method [5] that we previously used to solve the particular inverse problem. Among multiple ways to implement Tikhonov regularization, we adopted a MATLAB code developed by P.C. Hansen and modified to best suit our problem [6].

14. Supplementary videos

We attached necessary supplementary to describe the operation of the CCM. Please refer to the attached video files to watch the described videos.

Video S.1. 3D Volume rendering of the z-stack data obtained from a fluorescent bead imaging phantom using a two-photon microscope. Note the size of x-y plane shown is 200μm by 200μm, and not scaled to z-axis to aid visualization. Volume rendering and video editing was performed using a *ImageJ* with *3D Viewer* plugin.

Video S.2. 3D Volume rendering of the z-stack data obtained from a fluorescent bead imaging phantom using our computational cannula microscope. Note x-y plane (200μm x 200μm) is scaled to z-axis range of 2mm. Volume rendering and video editing was performed using a *ImageJ* with *3D Viewer* plugin.

Video S.3.(a)~(c). These videos show CCM images acquired at varying penetration depth while probe is inserting into the brain sample. We recorded CCM image at every 0.1mm from the surface to the 2mm deep inside the sample. Each video is the recording acquired while probing a spot located within (a) frontal lobe - left hemisphere, (b) frontal lobe - right hemisphere, and (c) parietal lobe - right hemisphere

Video S.4.(a)~(b). Each of these videos show two photon images obtained using two photon microscope from the same P3 post natal stage brain, from which the CCM images were acquired. Each video shows z-stack frames starting from the surface of the brain down to (a) 70μm and (b) 200μm under the surface. Images were acquired at 1μm axial step size. Field of view is 320μm by 320μm.

15. Comparison of CCM to other brain-imaging methods

Table S2: *Comparison of the performance of CCM against alternative techniques. References listed in the table are from the main text.*

Method	CCM [18]	Two photon [29]	Fiber bundle confocal [30]	GRIN microendoscope [31]
Measured Resolution	1μm	< ~2μm	3.3 μm	1μm
FOV	200μm diameter	620μm^2	330μm diameter	350μm diameter
Probe size	225μm diameter	Non-invasive	350μm diameter	500μm diameter
Scanning/Widefield	Widefield	Scanning	Scanning	Widefield

The resolution of CCM in Table 1 is based upon ref [18] in the main text, while that for two-photon microscopy is estimated from ref [29] in the main text.

References

[1] P. E. Debevec, J. Malik, "Recovering high dynamic range radiance maps from photographs," *ACM SIGGRAPH*, 31 (2008).
[2] Golub, G. H., & Reinsch, C. Singular value decomposition and least squares solutions. *Numerische mathematik* 14, 403-420 (1970).
[3] K. Pearson, Notes on regression and inheritance in the case of two parents. *Proc. R. Soc. A.* 58, 240-242 (1895)
[4] A. N. Tikhonov, et al., Numerical methods for the solution of ill-posed problems. Vol. 328. Springer Science & Business Media (2013)
[5] M. A. Seldowitz, J. P. Allebach, D. W. Sweeney, Synthesis of digital holograms by direct binary search., Applied Optics (1987).
[6] P. C. Hansen, Regularization tools: A Matlab package for analysis and solution of discrete ill-posed problems," Numerical algorithms (1994).
